# Supplementary material for: Differential role of cytosolic Hsp70s in longevity assurance and protein quality control
Source: PLoS Genet. 2021 Jan 11;17(1):e1008951. doi: 10.1371/journal.pgen.1008951 (PMC7822560; doi:10.1371/journal.pgen.1008951)
Supplement: S1 Materials and methods — (DOCX) [file pgen.1008951.s001.docx]

**Differential role of cytosolic Hsp70s in longevity assurance and protein quality control**

Rebecca Andersson, Anna Maria Eisele-Bürger, Sarah Hanzén, Katarina Vielfort, David Öling, Frederik Eisele, Gustav Johansson, Tobias Gustafsson, Kristian Kvint, Thomas Nyström

**SUPPLEMENTAL MATERIAL**

**SUPPLEMENTAL MATERIAL AND METHODS**

**Two‐dimensional polyacrylamide gel electrophoresis (2D‐PAGE).** Cell harvesting, protein extraction, and 2D‐PAGE were performed as described previously [1,2]. For the first dimension, strips ranging from pH 3–5.6 were used (GE Healthcare). The second‐dimension gels contained 7 % polyacrylamide. Total protein was visualized using silver staining. Staining was performed accordingly: fixation in 50% ethanol (v/v) plus 10% acetic acid for 2 h followed by washing in MilliQ water 3 × 20 min. Gels were sensitized in 500 ml DTT solution (5.2 mg/l) for 30 min. Subsequently, gels were incubated for 30 min in 500 ml silver nitrate solution (2 g/l). Gels were washed in 500 ml MilliQ water for 30 s, water poured off and 200 ml sodium bicarbonate (34.7 g/l) with formaldehyde (0.5 g/l) was added until a color development was observed. The solution was discarded and 500 ml fresh sodium bicarbonate with formaldehyde solution was added. Development of gels was stopped after approximately 5–6 min. Silver‐stained gels were scanned, aligned, and quantified with ImageJ. Ssa1–4 proteins were identified by visual matching with existing yeast 2‐D maps.

**Protein alignment.** Protein alignment of Ssa1 [3] and Ssa4 [4] were performed on amino acid sequences obtained from the S288C reference strain. Protein alignments were performed in Geneious 8.0.5 ® (Biomatters Ltd, New Zealand) by a global alignment using the Needleman-Wunsch algorithm [5] with BLOSUM62 cost matrix [6], and gap open penalty: 10, gap extension penalty: 0.5. Image was generated using Jalview 2 [7], conservation scores calculated as described by Livingstone and Barton [8].

**TABLE A: Strain list**

| **Figures** | **Name** | **Genotype** | **Reference** |
| --- | --- | --- | --- |
| 1A, D, E  2  3B, C  6A  S1A, B, D, F  S2A-E | BY4741/WT | Mat **a** *his3*Δ*1 leu2*Δ*0 met15*Δ*0 ura3*Δ*0* | [9] |
| 6A  S1A-D | **P_GPD_*SSA4*** | Mat **a** *his3*Δ*1 leu2*Δ*0 met15*Δ*0 ura3*Δ*0 natMX6:P_GPD_-SSA4* | This study |
| 1A, D, E  2  3B, C  4B, C  6A  S1A-D, F, H  S2A-C | *ssa1*Δ *ssa2*Δ | Mat **a** *his3*Δ*1 leu2*Δ*0 met15*Δ*0 ura3*Δ*0* *ssa1*Δ*::hph ssa2*Δ*::kanMX4* | [10] |
| 1A, D, E  2  3B, C  6A  S1A-D, F | *ssa1*Δ *ssa2*Δ **P_GPD_*SSA4*** | Mat **a** *his3*Δ*1 leu2*Δ*0 met15*Δ*0 ura3*Δ*0* *ssa1*Δ*::hph ssa2*Δ*::kanMX4*  *natMX6:P_GPD_-SSA4* | This study |
| 1B, C, F  3D, E  5  S1E, G  M1 | WT guk1-7-GFP | Mat **a** *his3*::pRS403*-guk1-7-EGFP::HIS3*  *leu2*Δ*0 met15*Δ*0 ura3*Δ | This study |
| 1B, C, F  3D, E  5  S1E, G  M2 | *ssa1*Δ *ssa2*Δ guk1-7-GFP | Mat **a** *his3*::pRS403-*guk1-7-EGFP::HIS3*  *leu2*Δ*0 met15*Δ*0 ura3*Δ  *ssa1*Δ*::hph ssa2*Δ*::kanMX4* | This study |
| 1B, C, F  3D, E  5  S1E, G  M3 | *ssa1*Δ *ssa2*Δ  **P_GPD_*SSA4*** guk1-7-GFP | Mat **a** *his3*::pRS403-*guk1-7-EGFP::HIS3*  *leu2*Δ*0 met15*Δ*0 ura3*Δ  *ssa1*Δ*::hph ssa2*Δ*::kanMX4*  *natMX6:P_GPD_-SSA4* | This study |
| 1B, C | WT gus1-3-GFP | Mat **a** *his3:*:pRS403-*gus1-3-EGFP::HIS3*  *leu2*Δ*0 met15*Δ*0 ura3*Δ | This study |
| 1B, C | *ssa1*Δ *ssa2*Δ *gus1-3*-GFP | Mat **a** *his3*::pRS403-*gus1-3-EGFP::HIS3*  *leu2*Δ*0 met15*Δ*0 ura3*Δ  *ssa1*Δ*::hph ssa2*Δ*::kanMX4* | This study |
| 1B, C | *ssa1*Δ *ssa2*Δ  **P_GPD_*SSA4*** gus1-3-GFP | Mat **a** *his*::pRS403-*gus1-3-GFP::HIS3*  *leu2*Δ*0 met15*Δ*0 ura3*Δ  *ssa1*Δ*::hph ssa2*Δ*::kanMX4*  *natMX6:P_GPD_-SSA4* | This study |
| 1B, C | Mca1-GFP | Mat **a** *his3*Δ*1 leu2*Δ*0 met15*Δ*0 ura3*Δ*0 MCA1-EGFP::HIS6* | [11] |
| 1B, C | *ssa1*Δ *ssa2*Δ Mca1-GFP | Mat **a** *his3*Δ*1 leu2*Δ*0 met15*Δ*0 ura3*Δ*0 MCA1-EGFP::HIS6*  *ssa1*Δ*::hph ssa2*Δ*::kanMX4* | [12] |
| 1B, C | *ssa1*Δ *ssa2*Δ  **P_GPD_*SSA4*** Mca1-GFP | Mat **a** *his3*Δ*1 leu2*Δ*0 met15*Δ*0 ura3*Δ*0 MCA1-EGFP::HIS6*  *ssa1*Δ*::hph ssa2*Δ*::kanMX4*  *natMX6:P_GPD_-SSA4* | This study |
| 3A | Ssa4-GFP Mca1-mRFP | Mat **a** *his3*Δ*1 leu2*Δ*0 met15*Δ*0 ura3*Δ*0 SSA4-GFP::HIS3 MCA1-mRFP::LEU2* | This study |
| 3A | *ssa1*Δ *ssa2*Δ Ssa4-GFP Mca1-mRFP | Mat **a** *his3*Δ*1 leu2*Δ*0 met15*Δ*0 ura3*Δ*0 SSA4-GFP::HIS3 MCA1-RFP::LEU2 ssa1*Δ*::hph ssa2*Δ*::kanMX4* | This study |
| S1E  M4 | *hsp104*Δ guk1*-7*-GFP | Mat **a** *his3*::pRS403-guk1-7-GFP*::HIS3*  *leu2*Δ*0 met15*Δ*0 ura3*Δ *hsp104*Δ::*URA3* | This study |
| S1E | *ssa1*Δ *ssa2*Δ *hsp104*Δ guk1-7-GFP | Mat **a** *his3*::pRS403-*guk1-7-EGFP::HIS3*  *leu2*Δ*0 met15*Δ*0 ura3*Δ *ssa1*Δ*::hph ssa2*Δ*::kanMX4 hsp104*Δ::*URA3* | This study |
| S1E | *ssa1*Δ *ssa2*Δ *hsp104*Δ **P_GPD_*SSA4*** guk1-7-GFP | Mat **a** *his3*::pRS403-*guk1-7-EGFP* *leu2*Δ*0 met15*Δ*0 ura3*Δ *ssa1*Δ*::hph ssa2*Δ*::kanMX4 hsp104*Δ::*URA3*  *natMX6:P_GPD_-SSA4* | This study |
| S1H | Ssa4-GFP | Mat **a** *his3*Δ*1* *leu2*Δ*0 met15*Δ*0 ura3*Δ *SSA4-EGFP::HIS3* | [11] |
| S1H | *ssa1*Δ *ssa2*Δ Ssa4-GFP | Mat **a** *his3*Δ*1* *leu2*Δ*0 met15*Δ*0 ura3*Δ  *ssa1*Δ*::hph ssa2*Δ*::kanMX4 SSA4-EGFP::HIS3* | This study |

**TABLE B: Plasmid list**

| **Figures** | **Name** | **Replicon** | **Promoter** | **Gene** | **Selection** | **Source** |
| --- | --- | --- | --- | --- | --- | --- |
| 3B, C  4B, C  S2A-E | pGFP-Hsp104 | CEN | *GAL1* | *EGFP-HSP104* | *HIS3* | [13] |
| 1B, C, F  3D, E  5  S1E, G  M1-4 | pguk1-7GFP | *-* | GPD | *guk1-7-EGFP* | *HIS3* | Per Widlund |
| 1B, C | pgus1-3GFP | *-* | GPD | *gus1-3-EGFP* | *HIS3* | Per Widlund |
| 2A | pΔssCL*** | CEN | Endogenous | Δ*ssCPY*-LEU2-myc* | *URA3* | [14] |
| 2B, C | pΔssCG*** | CEN | Endogenous | Δ*ssCPY*-EGFP-myc* | *URA3* | [15] |
| 4B, C  S2A-E | pRS415 | CEN | GPD | - | *LEU2* | [16] |
| 4B, C  S2A-E | p*SSA1* | CEN | GPD | *SSA1* | *LEU2* | This study |
| 4B, C  S2A-E | p*SSA4* | CEN | GPD | *SSA4* | *LEU2* | This study |
| 4B-D  S2A-E | p*NBDsw* | CEN | GPD | *NBD_SSA1_ssa4* | *LEU2* | This study |
| 4B-D  S2A-E | p*SBDsw* | CEN | GPD | *ssa4SBD_SSA1_* | *LEU2* | This study |
| 4B, C  S2A-E | p*CTDsw* | CEN | GPD | *ssa4CTD_SSA1_* | *LEU2* | This study |
| 5 | pSik1-RFP | CEN | GPD | *SIK1-mRFP* | *URA3* | [17] |

**SUPPLEMENTAL REFERENCES**

1. Maillet I, Lagniel G, Perrot M, Boucherie H, Labarre J. Rapid identification of yeast proteins on two-dimensional gels. J Biol Chem. 1996;271(17):10263-70.

2. Blomberg A. Use of two-dimensional gels in yeast proteomics. Methods Enzymol. 2002;350:559-84.

3. SGD. Ssa1: Saccharomyces genome database; 2018 [Available from: <https://www.yeastgenome.org/locus/S000000004>.

4. SGD. Ssa4: Saccharomyces genome database; 2018 [Available from: <https://www.yeastgenome.org/locus/S000000905>.

5. Needleman SB, Wunsch CD. A general method applicable to the search for similarities in the amino acid sequence of two proteins. J Mol Biol. 1970;48(3):443-53.

6. Henikoff S, Henikoff JG. Amino acid substitution matrices from protein blocks. Proc Natl Acad Sci U S A. 1992;89(22):10915-9.

7. Waterhouse AM, Procter JB, Martin DM, Clamp M, Barton GJ. Jalview Version 2--a multiple sequence alignment editor and analysis workbench. Bioinformatics. 2009;25(9):1189-91.

8. Livingstone CD, Barton GJ. Protein sequence alignments: a strategy for the hierarchical analysis of residue conservation. Comput Appl Biosci. 1993;9(6):745-56.

9. Brachmann CB, Davies A, Cost GJ, Caputo E, Li J, Hieter P, et al. Designer deletion strains derived from Saccharomyces cerevisiae S288C: a useful set of strains and plasmids for PCR-mediated gene disruption and other applications. Yeast. 1998;14(2):115-32.

10. Oling D, Eisele F, Kvint K, Nystrom T. Opposing roles of Ubp3-dependent deubiquitination regulate replicative life span and heat resistance. EMBO J. 2014;33(7):747-61.

11. Huh WK, Falvo JV, Gerke LC, Carroll AS, Howson RW, Weissman JS, et al. Global analysis of protein localization in budding yeast. Nature. 2003;425(6959):686-91.

12. Hill SM, Hao X, Liu B, Nystrom T. Life-span extension by a metacaspase in the yeast Saccharomyces cerevisiae. Science. 2014;344(6190):1389-92.

13. Tkach JM, Glover JR. Amino acid substitutions in the C-terminal AAA+ module of Hsp104 prevent substrate recognition by disrupting oligomerization and cause high temperature inactivation. J Biol Chem. 2004;279(34):35692-701.

14. Eisele F, Wolf DH. Degradation of misfolded protein in the cytoplasm is mediated by the ubiquitin ligase Ubr1. FEBS Lett. 2008;582(30):4143-6.

15. Medicherla B, Kostova Z, Schaefer A, Wolf DH. A genomic screen identifies Dsk2p and Rad23p as essential components of ER-associated degradation. EMBO Rep. 2004;5(7):692-7.

16. Sikorski RS, Hieter P. A system of shuttle vectors and yeast host strains designed for efficient manipulation of DNA in Saccharomyces cerevisiae. Genetics. 1989;122(1):19-27.

17. Sung MK, Huh WK. Bimolecular fluorescence complementation analysis system for in vivo detection of protein-protein interaction in Saccharomyces cerevisiae. Yeast. 2007;24(9):767-75.
