# Supplementary figures and images for: Differential role of cytosolic Hsp70s in longevity assurance and protein quality control
Source: PLoS Genet. 2021 Jan 11;17(1):e1008951. doi: 10.1371/journal.pgen.1008951 (PMC7822560; doi:10.1371/journal.pgen.1008951)

# SUPPLEMENTAL FIGURE 1

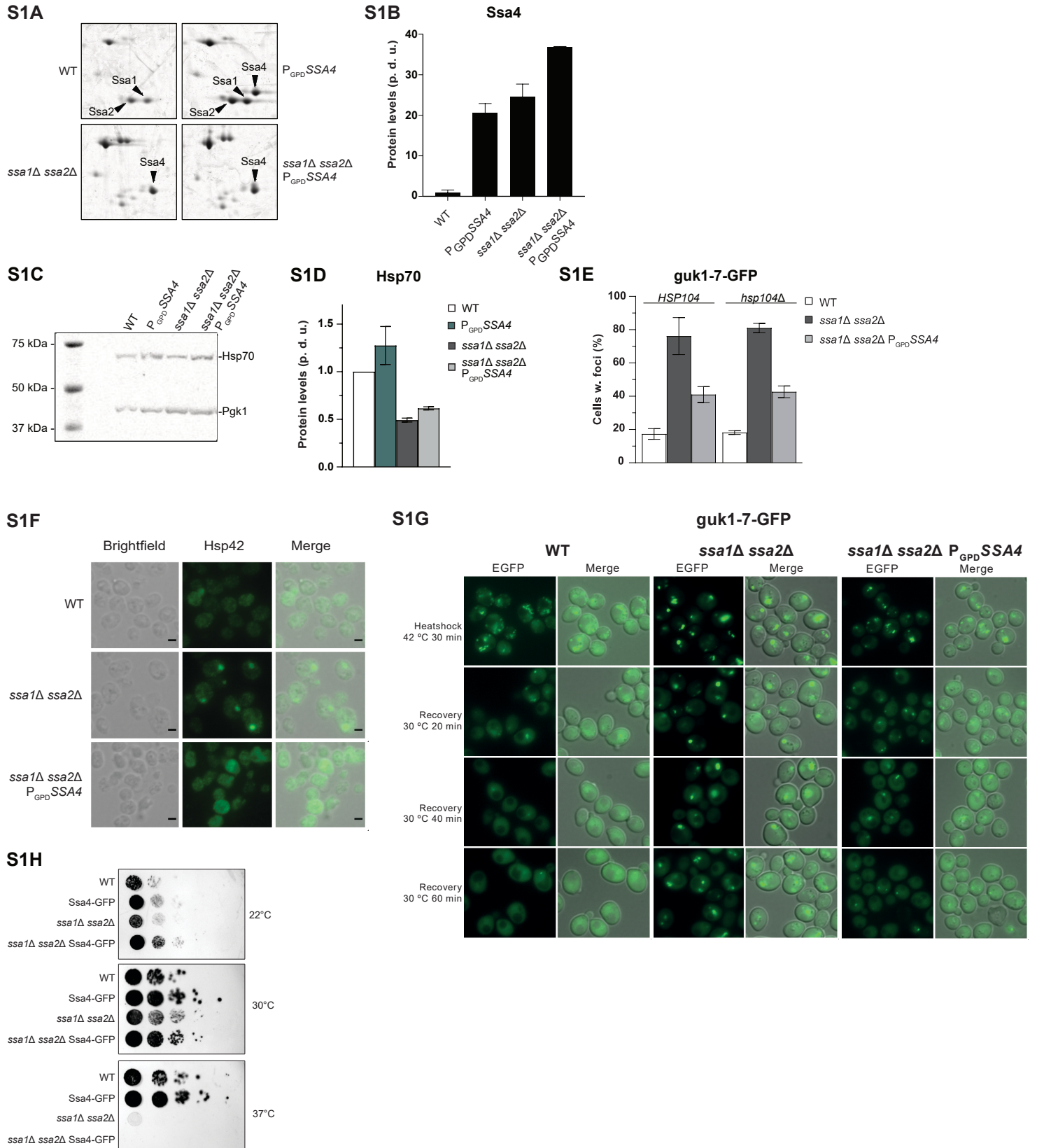

# SUPPLEMENTAL FIGURE 1

Supplement: S1 Fig — A) Cut-outs of silver stained 2-dimensinal polyacrylamide gels showing Ssa1, Ssa2 and Ssa4 in protein extracts from WT, PGPDSSA4, ssa1Δ ssa2Δ and ssa1Δ ssa2Δ PGPDSSA4 cells. Black arrows indicate the named proteins. B) Quantification the gels shown in A with respect to Ssa4 levels relative to Act1. WT is set to 1. Columns represent the mean of 2 (strains PGPDSSA4, ssa1Δ ssa2Δ and ssa1Δ ssa2Δ PGPDSSA4) or 3 (WT) biological replicates. Error bars: +/- S.D C) Representative western blot of SDS-PAGE gel with anti-Hsp70 antibody and anti-Pgk1 antibody as loading control. D) Quantification of immunoblotted SDS-PAGE gels seen in C with respect to total Hsp70 normalized against Pgk1 and with the WT protein level set to 1. Columns represent the mean of 2 biological replicates. Error bars: +/- S.D. E) Quantification of number of foci per cell of guk1-7-GFP in WT, ssa1Δ ssa2Δ and ssa1Δ ssa2Δ PGPDSSA4 with (left) or without (right) HSP104. Columns represent the mean of 4 biological replicates, n = 200. White bars: wild type (WT), dark grey bars: ssa1Δ ssa2Δ, and light grey bars: ssa1Δ ssa2Δ PGPDSSA4. Error bars: +/- S.D F) Fluorescence microscopy images of Hsp42 in WT, ssa1Δ ssa2Δ, and ssa1Δ ssa2Δ PGPDSSA4 by anti-Hsp42 antibody detection. Shown are images representative of 7 biological replicates. G) Fluorescence microscopy images of guk1-7-GFP cells after heat shock (42°C for 30 minutes) and during recovery at 30°C. Shown are images representative of three biological replicates. Images were generated as a maximum projection of one representative slice from a Z-stack using ImageJ 1.50i (Rasband, 1997) with 64-bit Java 1.8.0_77. H) Heat sensitivity test of WT and ssa1Δ ssa2Δ with or without GFP-tagged Ssa4. (PDF) [file pgen.1008951.s006.pdf]

## SUPPLEMENTAL FIGURE 2

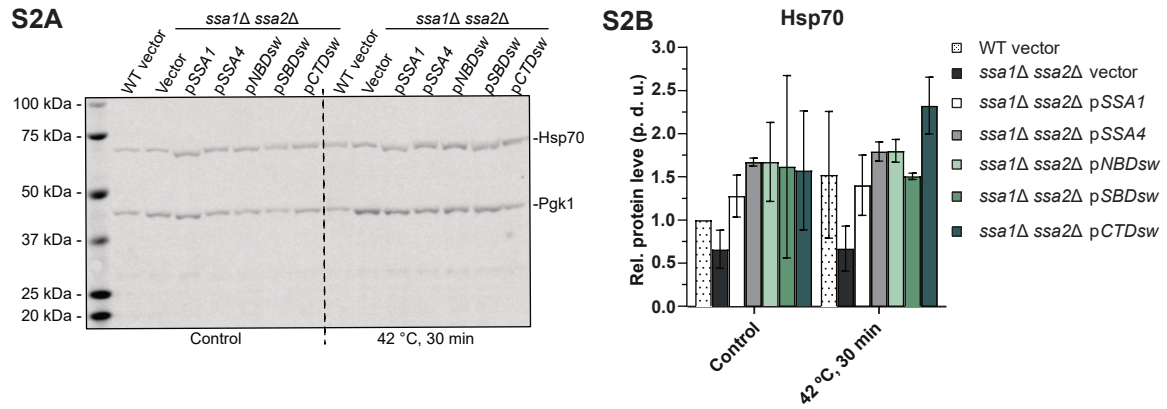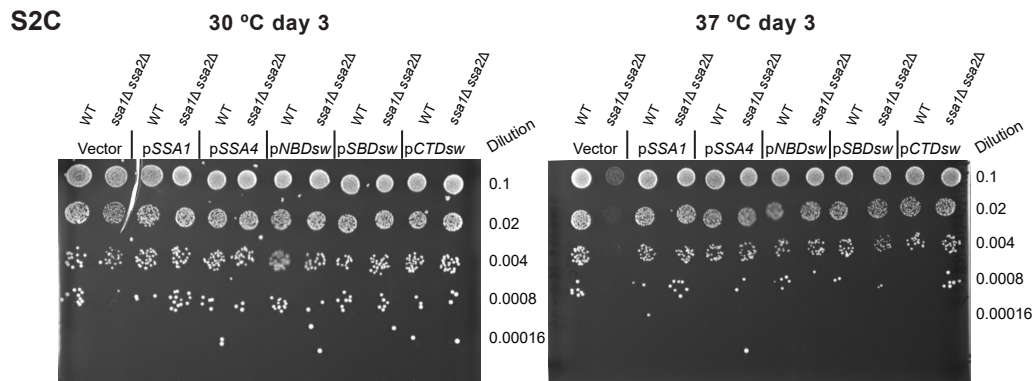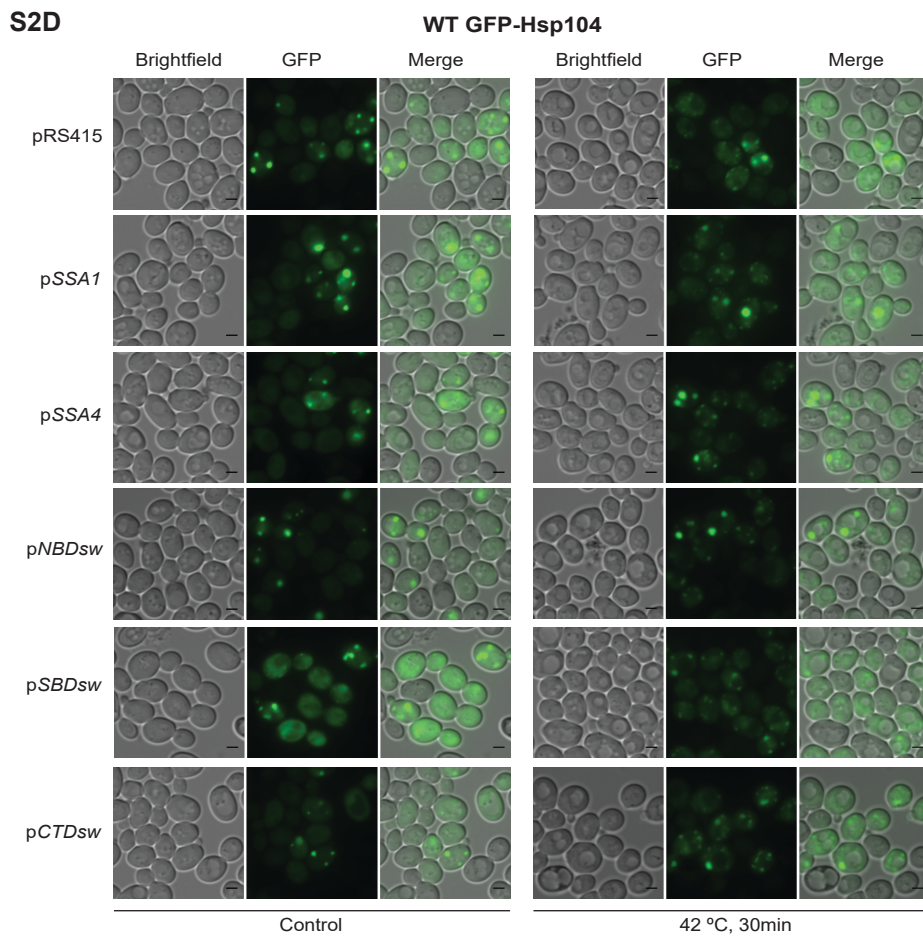

## SUPPLEMENTAL FIGURE 2

S2E

WT GFP-Hsp104

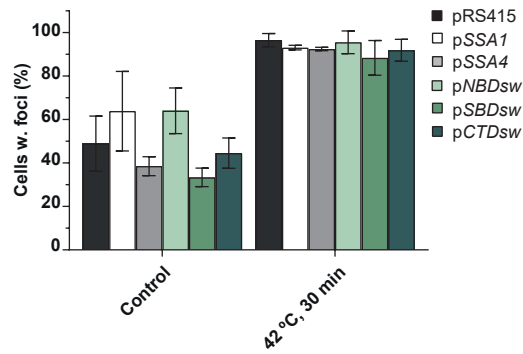

S2F

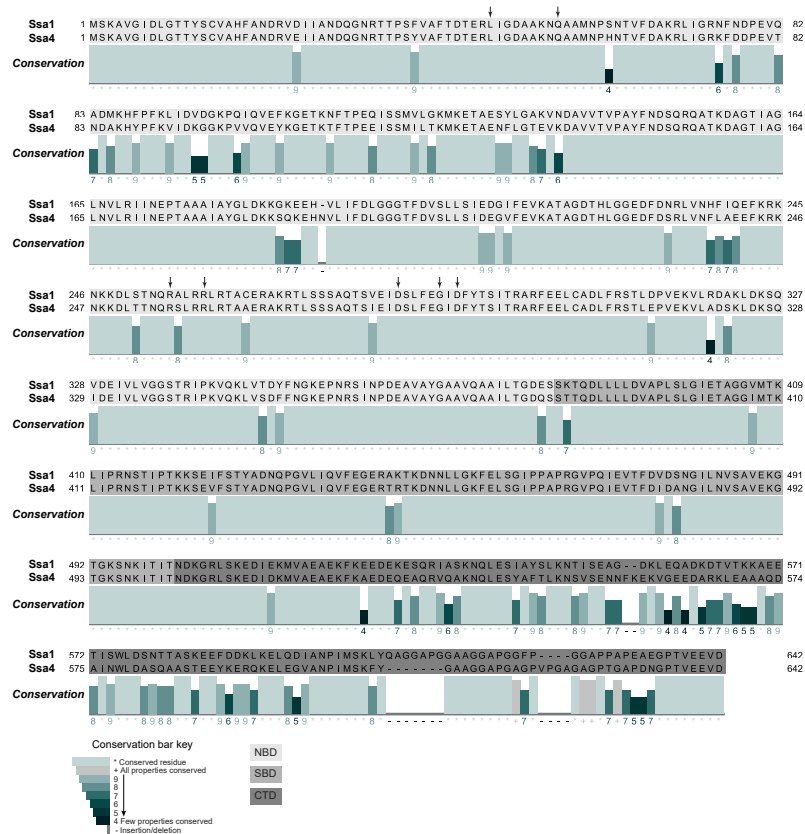

Supplement: S2 Fig — A) Representative western blot of SDS-PAGE gel with anti-Hsp70 antibody and anti-Pgk1 antibody as loading control. B) Quantification of immunoblotted SDS-PAGE gels shown in A with respect to total Hsp70. Values have been normalized to Pgk1 as loading control and the level in WT at steady state set to 1. Columns represent the mean of 2 biological replicates. Error bars: +/- S.D. White with black dots: WT pRS415, dark grey: ssa1Δ ssa2Δ pRS415, white: ssa1Δ ssa2Δ pSSA1, light grey: ssa1Δ ssa2Δ pSSA4, light green: ssa1Δ ssa2Δ pNBDsw, green: ssa1Δ ssa2Δ pSBDsw, and dark green: ssa1Δ ssa2Δ pCTDsw. C) Heat sensitivity test of wild type (WT), and ssa1Δ ssa2Δ with the empty vector (pRS415), wild type Ssa1 and Ssa4 (pSSA1 and pSSA4), and each of the three chimeric versions of Ssa4 (pNBDsw, pSBDsw and pCTDsw). The strains were serial diluted 1x10^-1^-1x10-5 and plated on synthetic medium agar plates for 3 days at the indicated temperatures. D) Fluorescence microscopy images of GFP-Hsp104 in wild type cells carrying the empty vector (pRS415), wild type Ssa1 and Ssa4 (pSSA1 and pSSA4), and each of the three chimeric versions of Ssa4 (pNBDsw, pSBDsw and pCTDsw), under exponential growth at 30°C (control) and heat shock (42°C, 30 minutes). E) Quantification of cells with foci from B. Columns represent the mean of 3 biological replicates, n = 200–209. Dark grey: pRS415, white: pSSA1, light grey: pSSA4, light green: pNBDsw, green: pSBDsw, and dark green: pCTDsw. Error bars: +/- S.D. F) Global alignment of Ssa1 and Ssa4. Conservation bar indicates the number of conserved physiochemical properties between the residues where * = conserved residue, + = all properties conserved, 9–1 = almost all to one property conserved,— = insertion/deletion/gap. Arrows (↓) mark residues previously identified as important in Ssa1-Hsp104 interactions [30]. Shaded boxes over residue codes indicate domain demarcations; light grey: NBD, grey: SBD, dark grey: CTD. (PDF) [file pgen.1008951.s007.pdf]
